# The Impact of Foreign Venture Capital Intervention on Venture Capital Innovation of Startup Entrepreneurs Using Propensity Score Matching Model

**DOI:** 10.3389/fpsyg.2021.750348

**Published:** 2021-12-09

**Authors:** Bei Han

**Affiliations:** School of Economics and Management, Northwest University, Xi’an, China

**Keywords:** PSM model, foreign venture capital, startup entrepreneurs, venture capital, enterprise innovation

## Abstract

The research expects to give full play to the role of venture capital in corporate innovation and enhance the development capability of enterprises. Based on Propensity Score Matching (PSM) model, the characteristics of venture capital and startup enterprises are analyzed, and the innovation of venture capital is discussed. Next, the PSM model is used to analyze the innovation of venture capital intervention in enterprises from risk probability intervention, probability evaluation, matching equilibrium validity test, matching results analysis, different venture capital, and different background risks. The results show that the difference of standardized mean is close to 0, which accords with the equilibrium test. The significant impact of venture capital intervention on the Number of Invention Patent Applications (NIPA) and Number of Utility Model Patent Applications (NUMPA) is 0.1 and 0.01, respectively. Venture capital intervention has a significantly positive impact on NIPA and NUMPA but has no significant positive impact on Number of Design Patent Applications (NDPA). The impact of joint venture capital intervention on the NIPA, NUMPA, and NDPA is 0.0874, 0.0635, and 0.1213, respectively. Hence, the intervention of joint venture capital can greatly promote the increase of Number of Patent Applications (NPA), especially, NIPA, and NUMPA. Compared with private venture capital, joint venture capital plays a greater role in promoting the growth of NPA and NIPA. Compared with private venture capital and foreign venture capital, national venture capital has a stronger innovation orientation and a longer investment cycle, which can greatly improve innovation performance, such as NIPA, while private venture capital and foreign venture capital have a less significant impact on enterprise innovation performance. The results demonstrate that the foreign capital sharing assessment based on the PSM model can be a good predictor of the performance of startups. It is hoped that the research results can provide a reference for the development of startups.

## Introduction

Innovation is the main driving force of economic development. With the intensification of economic and industrial chain globalization, the competition in the global market has shifted to the competition of global forces, which is technology-oriented (Elsafty et al., 2020; Mahfud et al., 2020). In the 1990s, with the rapid development of technological innovation of many high-tech companies in the United States, the rapid growth of the American economy and the growth of venture capital have played an important role in promoting technological innovation. Capital has cultivated numerous world-famous high-tech companies and helped the United States create the miracle of Silicon Valley (Belderbos et al., 2018; Janney et al., 2021). Facebook, Yahoo, and Amazon have become the representatives of high-tech enterprises. Today, if Chinese enterprises continue to adopt the low-tech processing of imported materials, their future development will be hindered. Chinese enterprises should turn to high value-added development with independent Intellectual Property Right (IPR) and enhance their independent Research and Development (R&D) capability (Luo et al., 2018). Therefore, China’s economic development model is facing a technological transformation. In recent years, Chinese enterprises have introduced and digested foreign advanced technology. However, Chinese enterprises can survive only through independent innovation and development of products, brands, and production technology with China’s independent IPR and develop better in international competition.

Startups (Startup enterprises) attach most importance to scientific and technological innovation activities, and their innovation and R&D are also the most active. They belong to the group of knowledge-intensive enterprises. At present, startups have become the key factor of China’s scientific and technological innovation ability and business transformation and development. However, with operational uncertainty, information asymmetry, and high agency costs, startups lack innovation and R&D funds, as well as relevant management and operation experience due to the capital constraints of traditional financing channels (Kohn, 2018; Seong and Kim, 2021). Hence, financial support should be provided for the business, as well as some value-added services and suggestions on operation, marketing, investment management, and strategic planning, that is, at different stages of the company’s development, the financing difficulties of innovative enterprises and innovative activities should be solved in the start-up stage through human resource allocation (Tang, 2020; Xu, 2020). In 2009, the Growth Enterprises Board (GEB) is launched in China, which has improved the capital market system and widened the outflow channels of venture capital, so that venture capital can exit smoothly. GEB provides a broad platform for the development of the venture capital industry. People are aware of the excellent performance of venture capital institutions in the high-tech entrepreneurial industry, while individual venture capital institutions have certain limitations in the capital, resources, and professional experience. Usually, projects and investment prospects are uncertain, so many venture capital institutions choose to invest in the same business, thereby forming a joint enterprise pattern. Under the common risk, start-ups can obtain professional knowledge and management, as well as corresponding value-added services. Consequently, investment risk can be reduced because of risk complimentary (Zhao et al., 2020; Zhang H. et al., 2021). Currently, the venture capital industry is developing rapidly, and scientific and technological innovation plays an ever-important role in economic development. Under this background, the relationship between venture capital and enterprise innovation has attracted the attention of researchers all over the world. The development of venture capital in China is late and immature, and the gap between that of foreign developed countries is still very large (Husain et al., 2021; Zhang X. W. et al., 2021). Particularly, the research of capital for enterprise innovation is of great practical significance. So far, few works of literature have taken into account both innovation contribution and innovation output, and few pieces of literature study the relationship between joint venture capital and the heterogeneity of its investment members and the characteristics of leading institutions. The impact of venture capital institutions on the innovation performance of enterprises is also many researchers concern. Here, whether joint enterprise investment will improve enterprise innovation level is studied through the empirical data of GEB to explore the impact of venture capital institutions on enterprise innovation performance.

Thereupon, the research takes startups as the research object, analyzes the factors of venture capital innovation through the model test, as well as the influence of foreign venture capital intervention in venture capital innovation of startup entrepreneurs. Innovatively, the impact of entrepreneurial venture capital innovation is studied by combining the Propensity Score Matching (PSM) model with the intervention of foreign venture capital in the venture capital of startup entrepreneurs. The purpose is to provide some references for startup entrepreneurs’ venture capital innovation consciousness.

## Related Concepts of Venture Capital

### Concept of Venture Capital

The concept of venture capital comes from the United States. Many famous organizations and associations have defined the concept of venture capital. So far, there is no unified definition of the concept of venture capital in academic circles. The American venture capital association gives the following definition: venture capital is equity capital (Duan et al., 2018; Awounou and Boufaden, 2020). Venture capitalists with industry expertise and skills prefer to invest in emerging companies with rapid growth. These companies are believed to have strong competitive potential in the market and can contribute to economic development. Venture capital is a kind of equity capital injected by venture capitalists into these companies. Its definition emphasizes the development characteristics of the invested enterprises.

The definition of venture capital given by the British Venture Capital Association emphasizes the development stage of the invested enterprises and is strictly distinguished from the different development stages of the enterprises (Hussain et al., 2020; Tian and Zhao, 2020). Venture capital is the capital obtained by enterprises in the entrepreneurial stage. In other words, venture capital is a special category of private capital and a kind of capital investment in the enterprise’s early development stage. Cheng Siwei, a famous Chinese scholar, said: Venture capital is a commercial investment behavior to trace the sources of capital risk. Venture capitalists invest in the R&D and high-tech fields of products with high-risk characteristics, especially, innovative enterprises based on high-tech (Joshipura and Joshipura, 2020; Li and Zhou, 2020). Additionally, investors and venture capitalists can provide professional advice and various value-added services. Specifically, they can serve the management and operation of the invested enterprises, promote the commercialization and mass production of innovative achievements, gain the initiative in market competition, and hope that the products can meet the needs of the public and succeed in the market, thereby obtaining a high return on investment and profits. Finally, venture capitalists will sell their equity to gain a return on their investment (Yang et al., 2020; Jiang and Martek, 2021). To sum up, here, venture capital is specifically defined: venture capital is an equity investment behavior, which emphasizes investing in innovative projects and startups to obtain high returns by helping enterprises to be listed or acquired on the stock exchange.

### Concept of Startups

Here, the startups are mainly studied. Startups are different from high-tech enterprises and Small and Medium-sized Enterprises (SMEs). They have the following three characteristics. Firstly, enterprise growth is in the entrepreneurial stage. Secondly, during their development, high growth and high-risk coexistence. Finally, startups emphasize innovation and are the extension of innovative enterprises. China’s GEB is the financing market for SMEs, high-tech enterprises, and startups to be listed on the main-board market, so many enterprises choose to be listed on the GEB (Delaney and Janzen, 2020; Wulandari, 2021). The factors of the enterprises’ innovation behaviors can be divided into external factors and internal factors: external factors include industry, region, political, cultural and legal environment, enterprise ownership, and the enterprise’s international environment; the internal factors include the size of the enterprise, the enterprise strategies, the internal governance system, the profitability of the enterprise, the capital structure, the characteristics of senior managers, and the participation of institutions.

### Venture Capital Innovation

Literature review shows that there has not been a unified conclusion about the impact of venture capital on enterprise innovation. Internationally, studies have been conducted on the post-investment management of venture capital, as well as the impact of venture capital on business performance and business innovation. Most existing research indicates that venture capital institutions can select companies to be invested in through their rich capital and sufficient experience in operations, management, and expertise, according to certain criteria, thereby reducing risks in venture capital (Letiagina and Malov, 2021). Through the uncertainty of financial contract investment, high-tech companies can carry out technological innovation activities to reduce operational and business uncertainty, venture capital institutions can improve the level of innovation. However, other literature has drawn the opposite conclusion that venture capital is not conducive to enterprise innovation, or is not conducive to innovation, and has nothing to do with innovation activities. Therefore, whether the intervention of venture capital organizations has a positive impact on enterprise innovation or a negative impact deserves further study (Palmer et al., 2020).

Additionally, there is plenty of literature involving the impact of venture capital on enterprise innovation, but little literature deeply discusses the relationship between joint venture capital and enterprise innovation. The individual venture capital in the form of joint venture capital can provide larger financial support for enterprises and allow enterprises to invest in R&D. Meanwhile, there is heterogeneity in knowledge, skills, and information networks among members of joint venture capital, so that venture capital members can benefit from professional knowledge, knowledge complementary, information sharing, and the overall evaluation of the projects and the shares (Li, 2020; Rodgers et al., 2020). Capitals are invested to significantly reduce investment risk and improve their project selection ability and value-added ability; technological innovation is promoted; on the other hand, members of venture capital joint enterprises can supervise and restrict each other (Filip, 2020; Lima et al., 2020). In this case, the probability that the innovation achievements of the invested enterprises are stolen by venture capital institutions is greatly reduced (Roh, 2020). Therefore, compared with individual venture capital enterprises, venture capital joint enterprises have a higher investment level in R&D, innovation, and development, a higher probability of innovation success, and more innovation achievements.

## Design Method of Foreign Venture Capital Intervention

### Model Design

Here, the PSM method is used to evaluate the impact of venture capital intervention on enterprise innovation performance, which can effectively avoid the deviation of sample selection. The basic principle of the PSM model is to construct comprehensive propensity scores according to the multidimensional characteristic variables of individuals and find individuals with similar scores or similar scores from the control group according to the comprehensive propensity score, thereby avoiding the matching difficulty caused by dimension. Further, the sample startups are divided into the experimental group and control group, with and without venture capital intervention, respectively. The propensity matching scores of startups are calculated by the PSM method, and then the enterprises with similar characteristics in the control group and the experimental group are selected by the Price-to-Sales (PS) value method. Afterward, the innovation performance of the experimental group and the control group is compared to study the impact of venture capital on the innovation performance of startups. When the PSM method is used to evaluate the impact of venture capital intervention on enterprise innovation performance, logit regression is first used to calculate the probability of introducing venture capital. The specific model is set as follows.


(1) $$P\left(A_{i}\right)=P\left({\text{WB}}_{i}=1|A_{i}\right)=\frac{\exp\left(\alpha A_{i}\right)}{1+\exp\left(\alpha X_{i}\right)}$$

**FIGURE 1:**
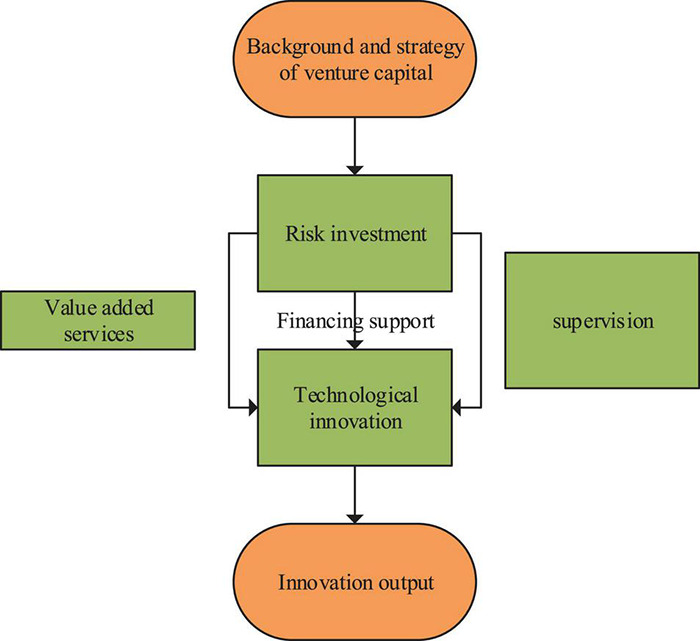
Structure of venture capital on technological innovation.

In (1), *P*(*A*_*i*_) represents the probability of venture capital intervening in enterprises, *A*_*i*_ indicates the multidimensional feature vector of enterprises, *P*() refers to the probability, and α indicates the coefficient corresponding to the multidimensional feature vector. WB = 1 represents the introduction of venture capital by the enterprise. Eq. (1) can calculate the enterprise propensity score, namely, the probability of venture capital intervention in the enterprise. Then, each company involved in venture capital corresponds to a company without introducing venture capital using the nearest neighbor matching method and radius matching method, and the efficiency difference between the processing group and the control group is evaluated using Attention (ATT) mechanism. Thereupon, this paper draws the structure diagram of venture capital intervention in venture capital innovation of startup entrepreneurs, as shown in Figure 1.

### Sample Selection and Data Processing

Firstly, the strategic objectives of Chinese startups are similar to venture capital. Secondly, in terms of the patent application, there is a certain lag in patent licensing for Chinese startups. Finally, for impact analysis of the venture capital introduction, the data of enterprise samples in recent years are too small to reflect the real situation of enterprise innovation performance. Here, Chinese startups are selected from authoritative databases, such as Wande and China Research Data Service Platform from November 2009 to November 2012 as the research object, along with the financial index data from September 2010 to September 2017.

Next, the characteristics of venture capital of each startup are found out, including background, investment strategy, technological innovation, and venture capital. For real estate, it is mainly manually organized through the Wande database, the national enterprise credit information system, visual inspection, and the official website of venture capital companies. According to the investment strategy of venture capital, venture capital is divided into independent venture capital and joint venture capital. Totally, 350 enterprises and 2,554 samples are collected here.

### Variable Definition

(1) Innovation performance: the Number of Patent Applications (NPA) is chosen to measure the innovation performance of the startups. Patents include three different types: invention patents, utility model patents, and design patents, of which the invention patents have incomparable advantages over utility models. Therefore, the NPA, Number of Utility Model Patent Applications (NUMPA), Number of Invention Patent Applications (NIPA), and Number of Design Patent Applications (NDPA) should all be considered for the innovation performance evaluation of the startups.
(2) Venture capital intervention: venture capital intervention is measured according to whether there are venture capital institutions among the major shareholders of the startups (WB). Venture capital is a dummy variable. If there is venture capital in the start-up enterprise, WB = 1, and otherwise, WB = 0. Additionally, the background and investment strategy of venture capital are defined. According to the background of venture capital, venture capital can be divided into national venture capital, private venture capital, and foreign venture capital. National venture capital is denoted by WB-G, and WB-G = 1 represents there is venture capital intervention in the startups, while WB-G = 0 means there isn’t. Similarly, private venture capital is denoted by WB-S, WB-S = 1 means there is venture capital intervention, and WB-S = 0 means there isn’t. Lastly, foreign venture capital is denoted as WB-W, WB-W = 1 means there is venture capital intervention, and WB-W = 0 means there isn’t. According to different investment strategies, venture capital can be divided into joint venture capital (WB-L) and independent venture capital (WB-D). Specifically, WB-L = 1 means there is joint venture capital intervention, and WB-L = 0 means there isn’t a joint venture capital intervention; WB-D = 1 means there is independent venture capital, and WB-D = 0 there isn’t an independent venture intervention.
(3) Variable matching: based on the above theoretical analysis, the PSM matching covariates of the experimental group and the control group read: Enterprise Scale (ES), ES is an important variable to measure enterprise-scale and affects the innovation performance of enterprises. ES is measured by the logarithm of total assets. Business Income (BI), BI refers to the enterprise income in the production and operation activities in a specific industry, which is calculated by the logarithm of the company’s main BI. R&D Investment (RDI), RDI affects the enterprise innovation performance and is also a basic variable that must be matched, measured by the proportion of R&D investment in the enterprise income. Current Ratio (CR), CR is the ratio of current assets and current liabilities and can measure the ability of an enterprise to turn current assets into cash to repay its liabilities before the maturity of short-term debts. Return On Assets (ROA), also known as asset profit margin, is the ratio of earnings before interest and tax to the enterprise’s average total assets in a specific period. Asset Growth Rate (AGR), AGR reflects the enterprise’s development capacity and capital accumulation capacity and is the ratio of the growth of the company’s total assets at the end of the year to the total assets at the beginning of the year. The increase in total assets this year is the difference between the year-end amount of total assets minus the amount at the beginning of the year. Equity Ratio (ER) is an important indicator to evaluate the enterprise’s capital structure, and it is measured by the ratio of total enterprise liabilities to total owner’s equity.

## Analysis of the Results of Foreign Venture Capital Intervention on Startups

### Venture Capital Intervention Probability Evaluation

**FIGURE 2:**
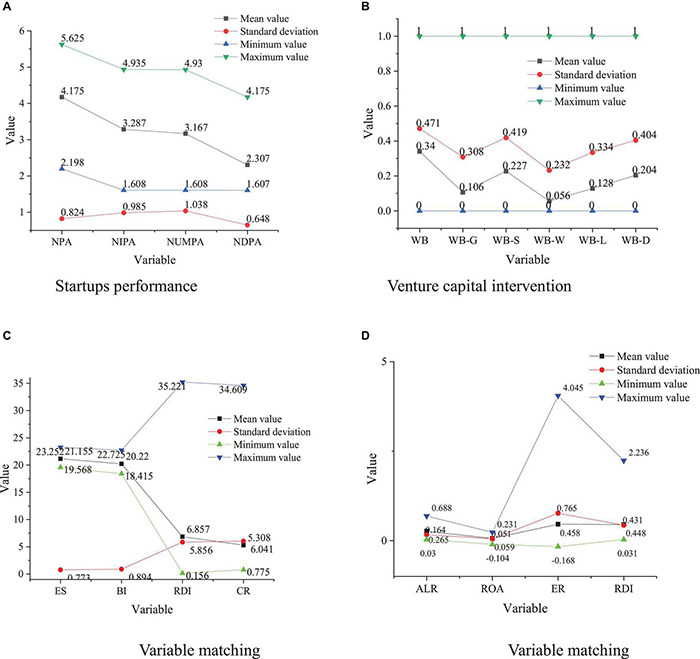
Variable description. **(A)** Startups performance. **(B)** Venture capital intervention. **(C)** Variable matching. **(D)** Variable matching.

**FIGURE 3:**
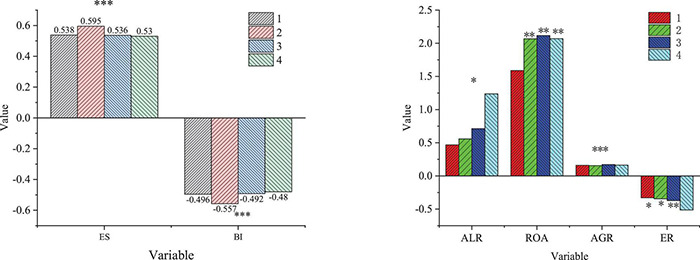
Results of logit model regression (^***^, ^**^, and * represents significance at the levels of 0.01, 0.05, and 0.1, respectively).

The probability of venture capital intervention in startups is estimated through logit regression. First, the sample startups are divided into the experimental group with venture capital intervention and the control group. Meanwhile, the Area Under the Curve (AUC) of Reciever Operating Characteristic Curve (ROC) and pseudo *R*^2^ are used for the selection of the control group. The specific results are shown in Figures 2, 3 and Table 1.

**TABLE 1:** Variable description statistics.

Variable	1	2	3	4
Industry variables	NO	YES	YES	YES
Pseudo *R*^2^	0.118	0.124	0.124	0.125
AUC	0.880	0.898	0.899	0.899
Sample	2,554	2,554	2,554	2,554

The descriptive statistical results of each variable can be obtained from Figure 2, including mean, standard deviation, minimum value, and maximum value. Figure 2 suggests the highest value of NPA in the startup table entry, the highest value of WB in venture capital intervention, the highest value of RDI in variable matching.

Figure 3 and Table 1 illustrate that the probability of venture capital intervention has a significantly positive relation with ES, ALR, ROA, AGR, and RDI, and has a significantly negative relation with BI and ER of the main enterprise, but has no relation with CR. The false 2 values of models 1, 2, 3, and 4 are between 0.118 and 0.125, and the AUC of ROC is between 0.880 and 0.899. The comparison of the false *R*^2^ and the AUC shows that model 3 is better than models 1, 2, and 4. Therefore, model 3 is used to calculate the probability of venture capital intervention to the enterprise, and then the innovation performance of enterprises with and without venture capital intervention is comparatively analyzed.

### Matching Equilibrium Validity Test

**FIGURE 4:**
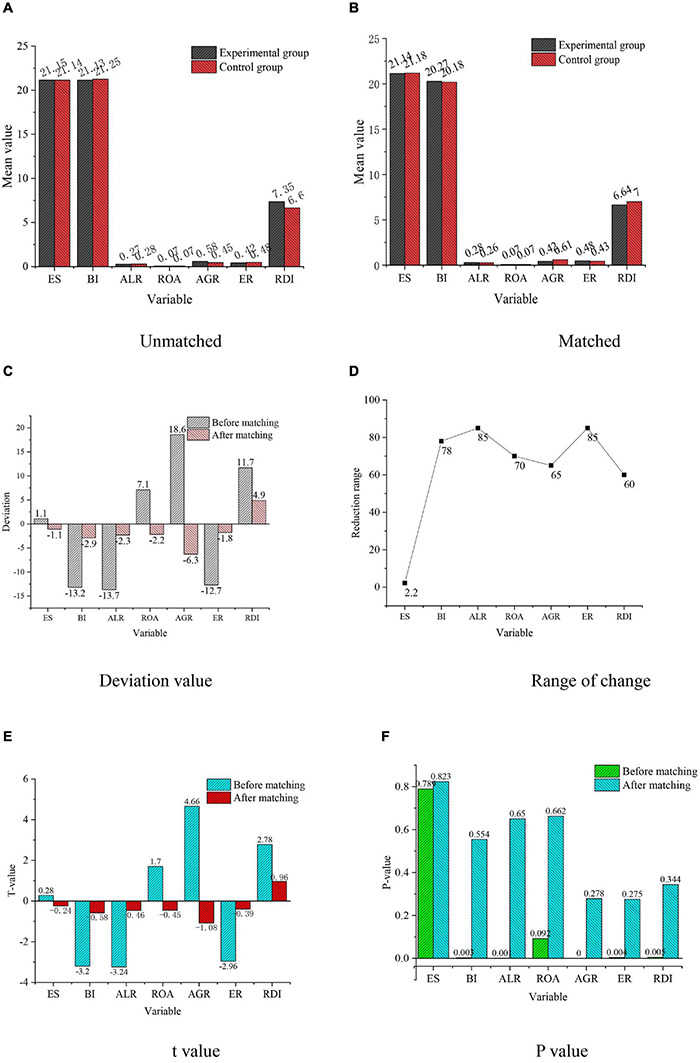
Test results of variable equilibrium. **(A)** Unmatched. **(B)** Matched. **(C)** Deviation value. **(D)** Range of change. **(E)** *t* value. **(F)** *P* value.

The results of the equilibrium test are shown in Figure 4.

Figure 4 demonstrates that the matching variables between the experimental group and the control group are equalized, and the *P* values of variables BI, ALR, ROA, AGR, ER, and RDI are less than 0.1 before matching. The hypothesis is that there is no significant difference between the experimental group and the control group. The experimental results show that *P*-value after matching is greater than 0.1, so there is no significant difference between the experimental group and the control group. Meanwhile, the *P*-value of variable ES is greater than 0.1, so there is no difference between the experimental group and the control group before and after matching. In short, after matching, all variables are similar, the non-equilibrium of all matched variables is significantly reduced, and the difference of standardized mean is close to 0, which indicates that the equilibrium test is met.

### Matching Result Analysis

**FIGURE 5:**
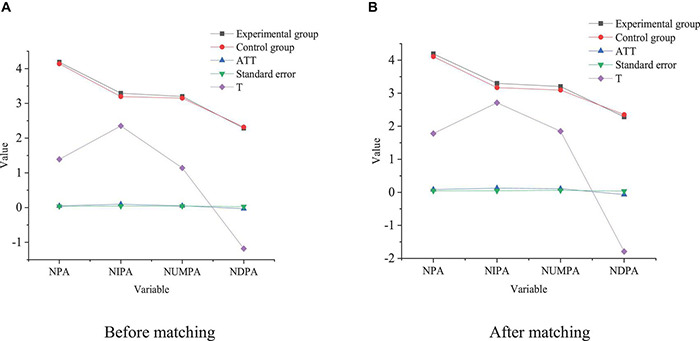
NNM. **(A)** Before matching. **(B)** After matching.

**FIGURE 6:**
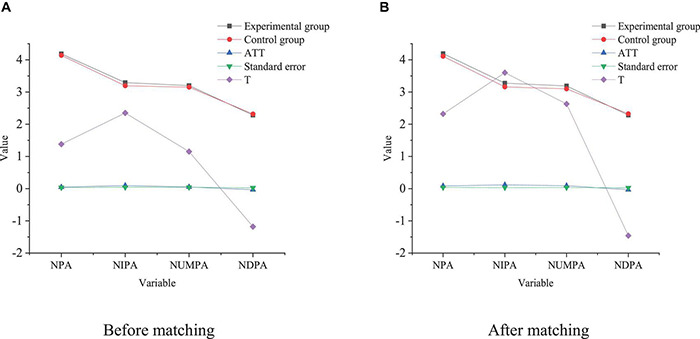
RM. **(A)** Before matching. **(B)** After matching.

Based on the equilibrium test, the NNM and RM are used to analyze the impact of venture capital intervention on enterprise innovation performance, as shown in Figures 5, 6, respectively.

Figure 6 illustrates that the impact of venture capital on the enterprise NPA is significantly different before and after matching, and venture capital can significantly promote the increase of the NPA of enterprises. The results of NNM show that the NPA of the experimental group and the control group are 4.1862 and 4.1384, respectively. Meanwhile, the ATT before matching is 0.0479, which has failed the significance test. The ATT of the experimental group and the control group after NNM are 4.107 and 4.1076, respectively. ATT after matching is significantly higher than that before matching. Hence, venture capital can play an important role in increasing the NPA, and the impact of venture capital intervention on NPA may be underestimated. Although venture capital can greatly promote the increase of the NPA, whether there is a different impact on the three kinds of patents remains to be explored. Here, the impact of venture capital on different types of patents is specifically analyzed. The results suggest that the impact of venture capital on the NIPA, NUMPA, and NDPA is 01268, 01171, and 01258, respectively, which is significant at the level of 0.01. Besides, the intervention effects of venture capital on the NIPA and NUMPA are 0109600912 and 00212, respectively, and the impact significance is 0.1 and 0.01, respectively. Compared with invention patents and utility model patents, the technical content of design patents is relatively low. The matching results imply that the value of the intervention effect minus NPA is negative, and matching is very important for NIPA and NUMPA, and the significance is closest to 0.1. By comparison, the significance level of RM indicates that the venture capital intervention has no obvious impact on NDPA, but the impact is not stable.

In summary, venture capital intervention has a significant positive impact on NIPA and NUMPA but has no significant positive impact on NDPA. The possible reason is that the sample is selected from startups, who have rapid growth of high-tech and pay more attention to innovative inventions and utility model patents. Although the R&D cycle is long, the results can bring higher expected returns to startups, so venture capital intervention plays an important role in improving their innovation performance.

### The Impact of Different Venture Capital on Innovation Performance

**FIGURE 7:**
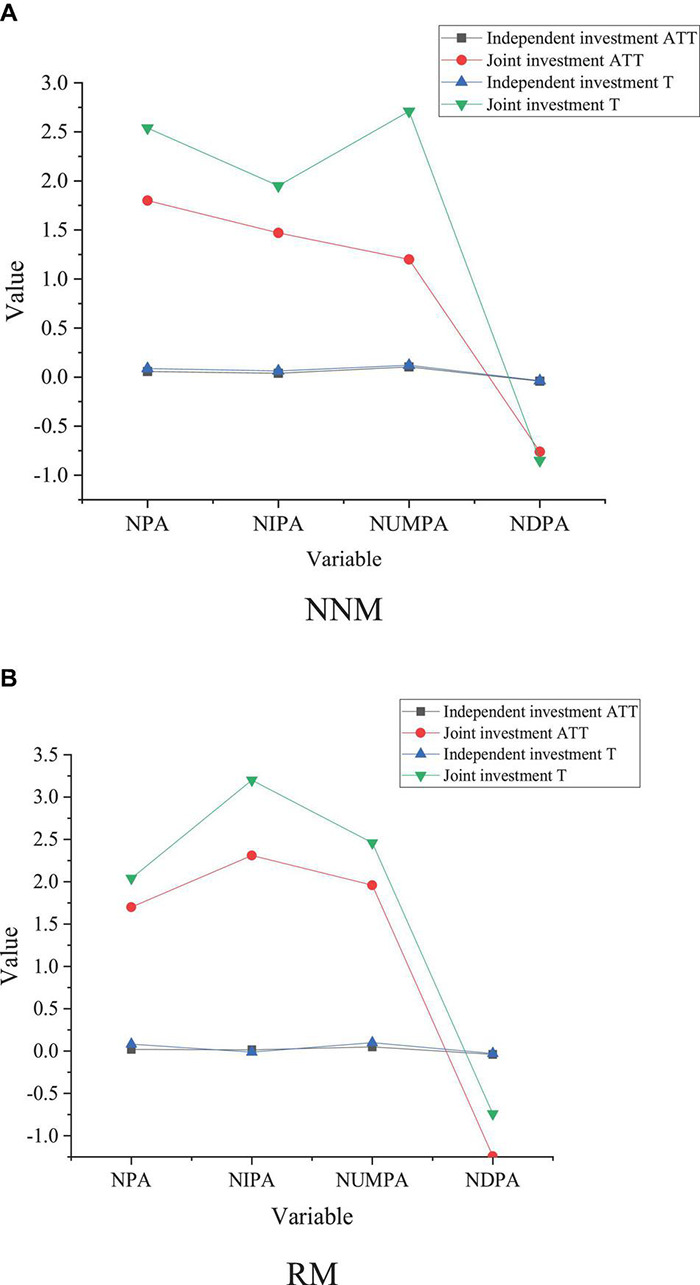
Impact of venture capital with the same strategy on startups performance. **(A)** NNM. **(B)** RM.

Afterward, the PSM method is used to empirically analyze the impact of venture capital on the innovation performance of enterprises with different investment strategies. The results are shown in Figure 7.

Figure 7 shows that independent venture capital has a significantly positive impact on the NPA. Under two different matching modes, the impact of the intervention of independent venture capital on the NPA and the number of ATT commercial patent applications are 0.0561, 0.0201, and 0.0916, respectively, which is significant at the level of 0.1. Only the RM method has passed the test by 0.05, and the other matching effects are not significant; the significance level of NUMP under three matching modes is 0.1038, 0.0450, and 0.0515, respectively. The other two methods have passed the significance test of 0.05 except for the closest matching. The intervention effects under three matching modes on the NDPA are –0.0409, –0.0389, and –0.0389, respectively, which have not passed the significance level test. It has a stable impact on three different types of patent applications.

According to the matching results of joint venture capital, joint venture capital has a significantly positive impact on enterprise innovation performance. The impact of joint venture capital intervention on the NIPA, NUMPA, and NDPA is 0.0874, 0.0635, and 0.1213, respectively, which is significant at level 0.1. RM and kernel matching show that the intervention of joint venture capital can significantly promote the increase of NPA. However, the intervention of joint venture capital on NDPA has failed to reach a significant level of 0.01 as the experimental design, which shows that the intervention of joint venture capital can greatly promote the increase of the NPA, especially, for NIPA and NUMPA. Compared with joint venture capital, ATT is more independent and plays a more important role in the promotion of NPA, the NIPA, and the NUMPA under joint venture capital, but it has no significant impact. Meanwhile, compared with venture capital, independent and joint venture capital is more conducive to technological innovation, This may be because, compared with independent joint venture capital joint involving multiple entities, ATT can better exercise its supervision function, management, and marketing and improve the innovation performance of the enterprise.

### The Impact of Venture Capital With Different Backgrounds on the Performance of Startups

**FIGURE 8:**
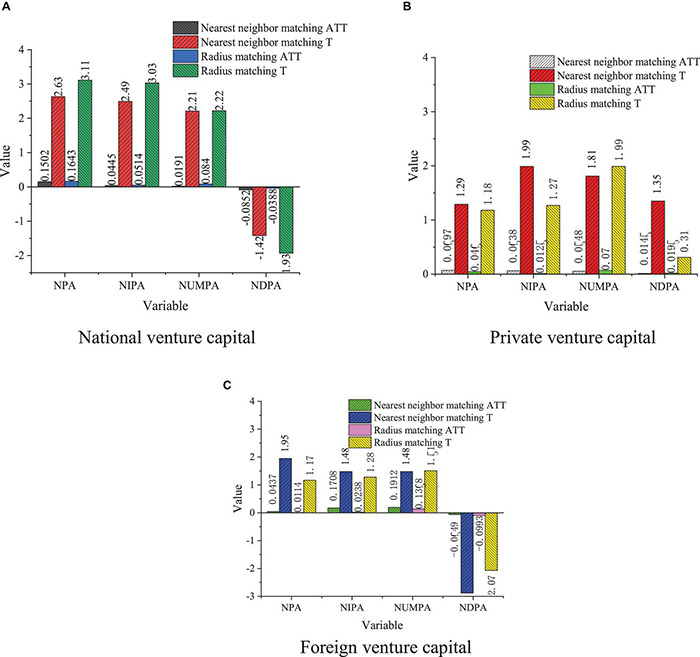
Background impact of venture capital on startups performance. **(A)** National venture capital. **(B)** Private venture capital. **(C)** Foreign venture capital.

The PSM method is used to analyze the impact of national, private, and foreign venture capital on enterprise innovation performance. The results are shown in Figure 8.

According to the NNM results, the intervention effect of national venture capital on the ATT of NPA, the NIPA, and the NUMPA are 0.5015, 0.0445, 0.0191, and 0.0852, respectively, which is significant at level 0.1 and is basically consistent with the detection results of the other two matching methods. Comparatively, the impact of private and foreign venture capital on enterprise innovation performance is not very significant. Apart from the private venture capital, under the three matching modes, the impact of venture capital intervention on the NUMPA are all significant at level 0.1, and the impact of venture capital on the other kinds of patents is not completely clear. Foreign venture capital has a negative impact on the NUMPA and has passed the test significance level of 0.05 The impact of venture capital of different backgrounds on technological innovation is different. Compared with private venture capital and foreign capital, national venture capital plays a more important role in promoting technological innovation because national venture capital usually pays attention to politics and creativity for a long time, and more investment time and major resources can be invested in activities, such as R&D. The impact of private venture capital and foreign venture capital enterprise innovation performance is not completely stable because private venture capital and foreign venture capital only consider short-term returns, which is unfavorable to their long-term investment.

## Conclusion

In order to improve the competitive ability of startups and enhance the resistance of firms to foreign investment risks, the research analyzes the impact of venture capital under different strategies and venture capital in different contexts on the performance of startups through propensity score estimation, matching validity test and matching result analysis. Here, a number of startups are analyzed from multiple perspectives and different conclusions are drawn. The impact of joint venture capital intervention on NIPA, NUMPA, and NDPA is 0.0874, 0.0635, and 0.1213, respectively. Joint ventures have a greater role in promoting the growth of NPA and NIPA compared to private venture capital. Compared to private venture capital and foreign venture capital, national venture capital has a stronger innovation orientation and longer investment cycle, which can significantly improve innovation performance, while private venture capital and foreign venture capital have no significant effect on the innovation performance of start-ups. By studying the impact of venture capital institutions on corporate innovation based on the micro perspective of corporate innovation, the research fits well with the development trend of national economic transformation, and reveals what kind of venture capital institutions can better play the role of incubating innovation. Moreover, it enriches the important proposition of venture capital and corporate innovation, and provides useful references for the development of startups. Furthermore, it also has some guiding significance for venture capital to improve incubation innovation. Here, the impact of venture capital and investment strategies on innovation performance of Chinese startups in different contexts is analyzed, and national venture capital is more capable of greatly improving innovation performance compared to private and foreign venture capital. However, the selection of the study sample is limited and not comprehensive due to the actual experimental conditions, which will be expanded in future studies. Meanwhile, a more comprehensive study can be conducted in terms of venture capital support function, financial investment, value-added services, and strategic investment in technological innovation.

## Data Availability Statement

The raw data supporting the conclusions of this article will be made available by the authors, without undue reservation.

## Ethics Statement

The studies involving human participants were reviewed and approved by Northwestern University Ethics Committee. The patients/participants provided their written informed consent to participate in this study. Written informed consent was obtained from the individual(s) for the publication of any potentially identifiable images or data included in this article.

## Author Contributions

The author confirms being the sole contributor of this work and has approved it for publication.

## Conflict of Interest

The author declares that the research was conducted in the absence of any commercial or financial relationships that could be construed as a potential conflict of interest.

## Publisher’s Note

All claims expressed in this article are solely those of the authors and do not necessarily represent those of their affiliated organizations, or those of the publisher, the editors and the reviewers. Any product that may be evaluated in this article, or claim that may be made by its manufacturer, is not guaranteed or endorsed by the publisher.
